# Self-sampling versus health care professional-guided swab collection for SARS-CoV-2 testing

**DOI:** 10.1007/s15010-021-01614-9

**Published:** 2021-05-10

**Authors:** Silvia Würstle, Christoph D. Spinner, Florian Voit, Dieter Hoffmann, Svenja Hering, Simon Weidlich, Jochen Schneider, Alexander Zink, Matthias Treiber, Roman Iakoubov, Roland M. Schmid, Ulrike Protzer, Johanna Erber

**Affiliations:** 1 Department of Internal Medicine II, School of Medicine, University Hospital Rechts der Isar, Technical University of Munich, Ismaninger Str. 22, 81675 Munich, Germany; 2 German Center for Infection Research (DZIF), Partner Site Munich, Munich, Germany; 3 Institute of Virology, School of Medicine, Technical University of Munich/Helmholtz Zentrum München, Munich, Germany; 4 Department of Dermatology and Allergology, School of Medicine, University Hospital Rechts der Isar, Technical University of Munich, Munich, Germany

**Keywords:** Self-sampling, Oropharyngeal, Swab, SARS-CoV-2, COVID-19

## Abstract

**Purpose:**

To evaluate the diagnostic reliability and practicability of self-collected oropharyngeal swab samples for the detection of SARS-CoV-2 infection as self-sampling could enable broader testing availability and reduce both personal protective equipment and potential exposure.

**Methods:**

Hospitalized SARS-CoV-2-infected patients were asked to collect two oropharyngeal swabs (SC-OPS1/2), and an additional oropharyngeal swab was collected by a health care professional (HCP-OPS). SARS-CoV-2 PCR testing for samples from 58 participants was performed, with a 48-h delay in half of the self-collected samples (SC-OPS2). The sensitivity, probability of concordance, and interrater reliability were calculated. Univariate and multivariate analyses were performed to assess predictive factors. Practicability was evaluated through a questionnaire.

**Results:**

The test sensitivity for HCP-OPS, SC-OPS1, and SC-OPS2 was 88%, 78%, and 77%, respectively. Combining both SC-OPS results increased the estimated sensitivity to 88%. The concordance probability between HCP-OPS and SC-OPS1 was 77.6% and 82.5% between SC-OPS1 and SC-OPS2, respectively. Of the participants, 69% affirmed performing future self-sampling at home, and 34% preferred self-sampling over HCP-guided testing. Participants with both positive HCP-OPS1 and SC-OPS1 indicating no challenges during self-sampling had more differences in viral load levels between HCP-OPS1 and SC-OPS1 than those who indicated challenges. Increasing disease duration and the presence of anti-SARS-CoV-2-IgG correlated with negative test results in self-collected samples of previously confirmed SARS-CoV-2 positive individuals.

**Conclusion:**

Oropharyngeal self-sampling is an applicable testing approach for SARS-CoV-2 diagnostics. Self-sampling tends to be more effective in early versus late infection and symptom onset, and the collection of two distinct samples is recommended to maintain high test sensitivity.

**Supplementary Information:**

The online version contains supplementary material available at 10.1007/s15010-021-01614-9.

## Introduction

The outbreak of severe acute respiratory syndrome coronavirus 2 (SARS-CoV-2) has caused an unprecedented burden on healthcare systems worldwide [1]. Diagnostic testing plays a pivotal role in controlling the coronavirus disease 2019 (COVID-19) pandemic, and polymerase chain reaction (PCR) remains the gold standard for detecting SARS-CoV-2 RNA that is extracted from the upper respiratory tract specimens collected by health care professionals (HCP) [2]. Evidence is growing that self-collected samples might serve as an acceptable and useful approach in SARS-CoV-2 testing [3–7]. Self-sampling may increase testing accessibility, preserve personal protective equipment, and reduce the exposure risk of medical staff and the public. The Food and Drug Administration has authorized antigen tests for over-the-counter use in December 2020. However, the test sensitivity is inferior in antigen tests and positive test results require confirmation by PCR. Thus, it is of utmost interest to develop self-sampling strategies for PCR testing. The efficacy and practicability of different self-sampling strategies have not been systematically investigated. In this study, we aimed to analyze the diagnostic reliability of self-collected oropharyngeal swabs (SC-OPS) in terms of sensitivity and practicability of use for the detection of SARS-CoV-2 infection. SARS-CoV-2 PCR from SC-OPS was compared to PCR from HCP-collected oropharyngeal swabs (HCP-OPS). To simulate shipping and assess the potential influences on diagnostic accuracy, a second SC-OPS sample was sent after 48 h of storage.

## Materials and methods

### Study population and participant recruitment

Participants were recruited from April to October 2020 from COVID-19 designated wards at the University Hospital rechts der Isar, Technical University of Munich, Munich, Germany. All participants were of legal age (≥ 18 years), hospitalized (not necessarily for COVID-19), and provided written informed consent (see Table 1 for details). Inclusion criterion was a positive SARS-CoV-2 PCR from an upper respiratory sample performed within the last 48 h before collection of the investigative swabs. The major exclusion criteria were physical or mental inability to provide informed consent and/or to perform self-sampling, as well as missing PCR results of SC-OPS1 or HCP-OPS. Severity of the disease was assessed at the day of sampling following the definitions of the severity of illness provided by the United States National Institute of Health (NIH) [8]. Briefly, the severity assessment covers five levels ranging from asymptomatic or pre-symptomatic infection to mild, moderate, severe, and critical illness.

**Table 1:** Baseline characteristics

Baseline characteristics	Median (range) or absolute and relative frequencies
Age; median (range)	59.5 (26–90)
Female sex; n/N (%)	24/58 (41%)
First positive SARS-CoV-2 PCR result 48 h before self-sampling; median (range)	2 (0–25)
Anti-SARS-CoV-2-IgG positive before or on the day of self-sampling; n/N (%)	20/58 (34%)
Free of COVID-19 symptoms; n/N (%)	7/58 (12%)
Interval symptom onset to self-sampling (days); median (range)	7.5 (0–31)
Number of indicated symptoms; median (range)	3 (0–6)
PCR test results	
HCP-OPS positive PCR; n/N (%)	43/58 (74%)
SC-OPS1 positive PCR; n/N (%)	38/58 (66%)
SC-OPS2 positive PCR; n/N (%)	37/57 (65%)
SC-ARS positive PCR; n/N (%)	1/23 (4%)
Viral load HCP-OPS (cps/mL); median (range)	2.9 × 10e3 (0–2.6 × 10e7)
Viral load SC-OPS1 (cps/mL); median (range)	2.6 × 10e3 (0–2.3 × 10e7)
Viral load SC-OPS2 (cps/mL); median (range)	9.6 × 10e2 (0–1.6 × 10e8)
Viral load SC-ARS (cps/mL); median (range)	0 (0–2.5 × 10e2)
Comparison of HCP-OPS and SC-OPS1	
HCP-OPS and SC-OPS1 PCR positive; n/N (%)	34/58 (59%)
HCP-OPS and SC-OPS1 PCR negative; n/N (%)	11/58 (19%)
HCP-OPS positive, SC-OPS1 PCR negative; n/N (%)	9/58 (16%)
HCP-OPS negative, SC-OPS1 PCR positive; n/N (%)	4/58 (7%)
HCP-OPS and SC-OPS1 PCR positive with viral load higher in HCP-OPS than SC-OPS1; n/N (%)	18/58 (31%)
HCP-OPS and SC-OPS1 PCR positive with viral load lower in HCP-OPS than SC-OPS1; n/N (%)	14/58 (24%)
Comparison of SC-OPS1 and SC-OPS2	
SC-OPS1 and SC-OPS2 PCR positive; n/N (%)	32/57 (56%)
SC-OPS1 and SC-OPS2 PCR negative; n/N (%)	15/57 (26%)
SC-OPS1 positive, SC-OPS2 PCR negative; n/N (%)	5/57 (9%)
SC-OPS1 negative, SC-OPS2 PCR positive; n/N (%)	5/57 (9%)
SC-OPS1 and SC-OPS2 PCR positive with viral load higher in SC-OPS1 than SC-OPS2; n/N (%)	17/57 (30%)
SC-OPS1 and SC-OPS2 PCR positive with viral load lower in SC-OPS1 than SC-OPS2; n/N (%)	14/57 (25%)

*HCP-OPS* Health Care Professional-collected oropharyngeal swabs sent for diagnostic without delay, *PCR* Polymerase Chain Reaction, *SC-OPS1* participant self-collected oropharyngeal swabs sent for diagnostic without delay, *SC-OPS2* participant self-collected oropharyngeal swabs sent for diagnostic after 48 h storage, *SC-ARS* Participant self-sampled anorectal swab sent for diagnostic without delay

### Sample size calculation

The sample size calculation was based on the assumption that statistical testing must detect a difference of ≥ 10% in sensitivity. Estimating a standard deviation of 0.25, which is the maximum range divided by 4, results in an effect size of 0.4 (*t* test, two-tailed, matched pairs). Setting alpha = 0.05, and beta = 0.8, 52 participants were included in this study, and an additional 20% were considered to account for potential dropouts, and hence, we included 63 participants.

### Sampling

Participants were asked to collect two oropharyngeal and facultatively one anorectal swab following two separate instructions with no further assistance (Supplementary Material 1). A single oropharyngeal swab was collected by an HCP. The sequence of collection of self-collected swabs and HCP-OPS was randomized at 1:1. HCP-OPS, facultative self-sampled anorectal swab (SC-ARS), and one of the self-collected oropharyngeal swabs (SC-OPS1) were immediately sent for diagnostic analysis following sampling. The other SC-OPS was stored at ambient temperature for 48 h (SC-OPS2) to assess the potential influence of a time delay from shipping in a prospective real-life setting with dry swaps. Participants were excluded from the analysis if SARS-CoV-2 PCR results from HCP-OPS or SC-OPS1 were not available.

### Diagnostic procedures

HCP-OPS were collected using eSwab 490CE (Copan, Brescia, Italy), containing viral transport media. SC-OPS1/2 were collected using FLOQswabs 552C 80 mm (Copan, Brescia, Italy, no transport media). UTM 305C swabs (Copan, Brescia, Italy), which contain viral transport media, were used for the SC-ARS.

The mSample Preparation System DNA kit identical to the Promega Maxwell^®^ Viral Total Nucleic Acid Extraction Kit (Promega, Medison, WI, USA) was used for nucleic acid extraction following a standard protocol on an m2000sp device for RNA and DNA extraction (Abbott, Wiesbaden, Germany), SARS-CoV-2 RT PCR was performed using SARS-CoV-2_N1 and SARS-CoV-2_N3 primer and probe sets for amplification on an ABI 7500 real-time PCR cycler (Thermofisher Scientific, Darmstadt, Germany) following the protocol of the Division of Viral Diseases, National Center of Immunization and Respiratory Diseases, Centers for Disease Control and Prevention Atlanta, USA [9]. Quantitative SARS-CoV-2 PCR results were calculated with a standard curve generated from 10e6, 10e4, 10e2, and 10e1 standards. The standard consists of a complete, cloned capsid gene and was produced in house. Results in copies per milliliter (copies/mL) represented viral loads in swab samples. Viral loads less than the lowest standards of 10 copies per reaction cannot be quantitated and thus are labeled < 500 cps/mL. For statistical evaluation of such samples, 250 cps/mL was assumed as viral load. Self-sampled swabs were analyzed individually to assess the effect of 48 h of storage at room temperature and delayed PCR testing. To theoretically investigate the pooling of two samples, results from HCP-OPS1 and -2 were combined and were rated positive if SARS-CoV-2 RNA was detected in either of the samples.

### Sensitivity and accuracy

To calculate sensitivity, the results obtained from participants with at least one positive SARS-CoV-2 PCR test from HCP-OPS, SC-OPS1, or SC-OPS2 were considered true-positive, giving no false-positive test results (see Table 2 for details). If SARS-CoV-2 PCR remained negative in all swabs, the results were defined as true-negative.

**Table 2:** Calculation of sensitivity

	Abbreviation and formula	HCP-OPS	SC-OPS1	SC-OPS2	Pooled SC-OPS1 + SC-OPS2
True-positive	Tp	43	38	37	43
True-negative	Tn	9	9	9	9
False-positive	Fp	0	0	0	0
False-negative	Fn	6	11	11	6
Accuracy	(Tp + Tn)/(Tp + Tn + Fp + Fn)	90%	81%	81%	90%
Sensitivity	Tp/(Tp + Fn)	88%	78%	77%	88%

In total, 49 tests were considered positive, since at least one of the PCR tests (HCP-OPS, SC-OPS1, or SC-OPS2) revealed a positive result. Results from SC-OPS1 and SC-OPS2 were retrospectively combined for theoretical pooling of two distinct tests (see Methods section for further details)

*HCP-OPS* Health Care Professional-collected oropharyngeal swabs sent for diagnostic without delay, *SC-OPS1* participant self-collected oropharyngeal swabs sent for diagnostic without delay, *SC-OPS2* participant self-collected oropharyngeal swabs sent for diagnostic after 48 h storage

### Evaluation of practicability with standardized closed and qualitative open questions

Potential advantages or problems in the performance of self-collection, as well as the preference for self-sampling versus HCP-guided swab collection, were assessed using a questionnaire with three closed, one half-closed, and one half-open questions (Supplementary Material 2). Since all participants had answered Question 1, we assumed that not checking "no problems" or "gag reflex" corresponded to a "no" as an answer, respectively. Handwritten responses were transcribed and translated to English (Supplementary Material 3).

### Statistical methods

The distribution of quantitative and qualitative data is presented as absolute and relative frequencies or medians (range), respectively. Exact 95% confidence intervals and exact binomial tests were calculated for the probability of concordance. Cohen’s *κ* was used for interrater reliability. Bland–Altman plots depict agreements between HCP-guided swab and self-sampling. Fisher’s two-sided exact test or Pearson’s Chi-squared test were performed on categorial variables and Wilcoxon’s rank-sum test on quantitative parameters. In addition to this univariable analysis, a multivariable analysis was performed by binary logistic regression. Statistical hypothesis testing was performed on two-sided exploratory 0.05 significance levels. Confidence intervals are given in square brackets. All statistical analyses were performed using R Studio version 4.0.2 (R Foundation for Statistical Computing, Vienna, Austria).

## Results

### Baseline characteristics

Of the 63 participants who were enrolled, 5 were excluded due to missing SC-OPS1 or HCP-OPS results. One participant with missing PCR from SC-OPS2-PCR was kept for analysis. The median age of the 58 remaining participants was 59.5 years (range 26–90 years) and 24 (41%) were female. Of the participants, 12% were asymptomatic and all others reported up to six different symptoms related to COVID-19. The median time from symptom onset to study enrollment was 7.5 days (range 0–31). 8.6% (*n* = 5) of the participants had asymptomatic or pre-symptomatic SARS-CoV-2 infections at the day of sampling and 15.5% (*n* = 9) suffered from mild illness, while the majority of participants went through moderate (*n* = 25, 43.1%) or severe (*n* = 19, 32.8%) disease as defined by the US National Institutes of Health [8].

### Sensitivity and accuracy

For HCP-collected samples, the results of SARS-CoV-2 PCR were found to be positive for 43 samples (74%). The SC-OPS1 and SC-OPS2 samples revealed 38 (66%) and 37 positive results (65%), respectively. In total, 49 participants tested positive for SARS-CoV-2 for at least one sample. Facultative SC-ARS was collected only by 23 participants (40%), one of which tested positive for SARS-CoV-2 (4%), resulting in a test sensitivity of 6%. The test sensitivity was estimated to be 88% for HCP-OPS, as opposed to 78% and 77% for SC-OPS1 and SC-OPS2, respectively (see Table 2 for details). We hypothesized that combining both results from SC-OPS could increase diagnostic sensitivity. When results from self-sampling were considered positive if SARS-CoV-2 PCR from SC-OPS1 and/or SC-OPS2 yielded positive results; the pooled estimated sensitivity resulted in 88% (Table 2). The accuracy for HCP-OPS, SC-OPS1, SC-OPS2, and the pooled SC-OPS1 and SC-OPS2 results was found to be 90%, 81%, 81%, and 90%, respectively.

### Testing for concordance

Binominal testing resulted in a concordance probability of 77.6% between HCP-OPS and SC-OPS1 (64.7–87.5%, *p* < 0.0001). Cohen’s *κ* was found to be 0.47 [0.22–0.73], suggesting a moderate strength of agreement [8]. For SC-OPS1 and SC-OPS2, concordance probability was estimated to be 82.5% [70.1–91.3%, *p* < 0.0001] and Cohen’s κ was found to be 0.62 [0.40–0.83], indicating a substantial strength of agreement [10]. Viral load of the positive sample was < 500 cps/ml in 5 of 10 patients with different qualitative results of SC-OPS1 and SC-OPS2. 3 of 5 patients with contradictory SC-OPS1 and SC-OPS2 results showed SC-OPS1 positive and SC-OPS2 negative results, and 2 of 5 patients had SC-OPS1 negative and SC-OPS2 positive results.

The median viral load was found to be 2.9 × 10e3 cps/mL for HCP-OPS (range 0–2.6 × 10e7 cps/mL), 2.6 × 10e3 cps/mL (range 0–2.3 × 10e7 cps/mL) for SC-OPS1, and 0.96 × 10e2 cps/mL (range 0–1.6 × 10e8 cps/mL) for SC-OPS2, with a decrease of 63% median viral load in SC-OPS2 compared to SC-OPS1.

The Bland–Altman plots show the difference in viral load between HCP-OPS and SC-OPS1 as well as SC-OPS1 and SC-OPS2, and depict the comparison of physician-guided testing and self-sampling as well as self-sampling with and without simulation of shipping at 48 h, respectively, on a continuous level including 95% confidence intervals (Supplementary Fig. 1). The discrepancy between the two compared methods is around zero, with a wide range of limits of agreement as the average viral load increases.

### Assessment of participants

Evaluation of questionnaires revealed that 48% of participants did not experience any challenges during self-sampling, and 48% of participants reported that a gag reflex was triggered (Supplementary Table 1). Of the participants, 34% claimed to prefer self-sampling over HCP-guided testing, while 22% prospectively preferred HCP-guided testing, and 41% were indifferent. In total, 69% of participants concluded that self-sampling at home would be conceivable in the future (Supplementary Table 1). See Supplementary Material 3 for the translated answers to the half-open questions.

Preference for testing was not found to correlate with age, sex, or positive SC-OPS1 results. The assumed conceivability of self-sampling was not found to correlate with the positive SC-OPS1 results. Of note, all participants with negative SC-OPS1 but positive HCP-OPS1 indicated that future self-sampling would be conceivable. Interestingly, participants with both positive HCP-OPS1 and SC-OPS1 (34/58; subgroup), indicating no problems during self-sampling had more differences in viral load levels (cps/mL) between HCP-OPS1 and SC-OPS1 than participants who indicated problems (*p* = 0.07416). Similarly, higher differences between HCP-OPS1 and SC-OPS1 values were found in participants indicating no gag reflex (*p* = 0.0556). Notably, in participants with concordant SARS-CoV-2 PCR in HCP-OPS and SC-OPS1, the divergence of viral load was higher when no gag reflex (*p* = 0.056) during self-sampling was reported. Remarkably, SC-OPS1 was never false-negative in participants feeling more secure or less frightened with HCP-OPS compared to self-sampling.

### Predictive factors

Age and sex were not found to correlate with positive SC-OPS1 results (*p* = 0.868 and 0.782, respectively). Men showed slightly higher differences between HCP-OPS and SC-OPS1 viral load than women (mean 2.1 × 10e6 cps/mL for men, mean 1.2 × 10e5 cps/mL for women; *p* = 0.301).

As expected, symptom onset more than 7 days before presentation was found to be a predictor for negative results for SC-OPS1 (*p* = 0.001) and concordant negative test results in HCP-OPS and SC-OPS1 (*p* = 0.038). Furthermore, the prevalence of anti-SARS-CoV-2-IgG in routine diagnosis obtained before or on the day of self-sampling showed a significant correlation with negative PCR results from SC-OPS1 (*p* = 0.004) or both negative results from HCP-OPS and SC-OPS1 (*p* = 0.005), as well as less differences in viral load. Contradictory results from HCP-OPS and SC-OPS1 were accompanied by a lower median viral load of SC-OPS1 (*p* = 0.001).

In the multivariable logistic regression analysis, subjective symptom onset of more than 7 days remained significant for negative test results from SC-OPS1 and both HCP-OPS and SC-OPS1 after adjusting for subjective gag reflex (Supplementary Table 2).

## Discussion

In this study, we aimed to assess the sensitivity, feasibility, and acceptance of self-collected oropharyngeal swabs for the detection of SARS-CoV-2 infection.

Previous studies have compared the sensitivities of the self-collected tongue, nasal, or mid-turbinate swabs to HCP-collected nasopharyngeal swabs [3, 4]. Furthermore, combined self-collected specimens (OPS plus mid-turbinate or OPS plus nasal swab) were compared to HCP-collected oropharyngeal and mid-turbinate or nasopharyngeal swabs, respectively [5, 6, 11]. The sensitivity of SC-OPS as compared to HCP-OPS has not been investigated to date.

In our cohort, the sensitivity of a single SC-OPS was inferior when compared to that of HCP-OPS (78% versus 88%). Similar ranges for sensitivity and inferiority have been reported in previous studies, although comparability is hampered by the fact that specimens were collected from participants with symptoms suggestive of COVID-19 in these studies, and the definitions of true- and false-positive results were different [3, 4, 6, 11]. Qualitative results between SC-OPS1 and 2 differed for 10 patients, which might be based on random distribution of molecules in low concentrated samples or a less vigorous approach in one of the self-collected samples. Intriguingly, combining the PCR results from both the self-collected swabs taken at the same time (SC-OPS1 and 2) resulted in an estimated sensitivity that was comparable to HCP-OPS, suggesting that two samples should be collected in a future self-sampling scenario. In contrast to previous studies where multiple testing from different anatomic sites was compared to HCP-collected samples, multiple sampling from one anatomic site was performed and appears to be efficient. This is an important finding, because sampling from one site facilitates the performance of self-sampling. The pooling of both swabs and performing one PCR would reduce resources in the face of limited testing capacities and should be further investigated.

We observed markedly decreased viral load in self-collected samples that were sent for SARS-CoV-2 PCR testing after 48 h of dry storage at room temperature, possibly due to underlying degradation of viral RNA. However, the sensitivities of SC-OPS1 and SC-OPS2 were similar and concordance between both tests was substantial, suggesting that shipping and consequently delayed testing in a self-sampling setting at home would not affect the test results. This is in line with data reported by Roger et al. who found increasing Cycle Threshold (CT) values over time of sample storage in saline without impact on the qualitative interpretation [12]. Theoretically, use of viral transport media could be beneficial in case of low viral load. However, previous studies did not show differences in sensitivity of dry swabs compared to liquid transport media [13–15]. Aside, use of liquid transport media complicates handling of non-healthcare professional users and transport. Moreover, higher viral loads could be expected shortly after symptom onset, while degradation of viral load may not significantly influence PCR results. As we aimed to design a pragmatic and non-professional approach for this study, a dry swab approach was chosen. To study the influence of degradation of RNA with this method, a shipping time of 48 h was simulated.

The practicability of self-sampling is a key factor in the acceptance and success of self-sampling strategies. Evaluation of questionnaires revealed that more than one-third of the participants preferred self-sampling over HCP-guided sampling. In total, almost two-thirds of the participants reported that they would perform self-sampling at home. Several participants appreciated that self-sampling would reduce the exposure risk of HCPs. Others claimed that self-sampling is more comfortable. Although a bias due to the introductive conversation for enrollment cannot be excluded, our data suggest that participants would accept self-sampling.

Next, we aimed to determine predictive factors for positive or negative SARS-CoV-2 PCR results for the self-collected specimens. Age, sex, and subjectively perceived capability to perform self-sampling are not correlated with successful detection of SARS-CoV-2 infection, which supports possible future self-sampling for different cohorts. We note that several patients of older age were cognitively or physically not able to consent in participation in this study resulting in a median age of 59.5 years. Thus, implementation of self-sampling strategies might be limited in the elderly, but could be offered to all people physically and cognitively able to collect swabs. Gagging was triggered in almost 50% of cases; however, the occurrence of this reflex did not result in a decreased positivity rate. Given an overall higher viral load in the samples of participants reporting problems such as gagging or coughing, we hypothesize that provoking these phenomena may indicate correct sampling performance. Participants should be encouraged to perform self-sampling, since the fear of failure was mentioned most frequently in participants who preferred HCP-guided swab collection to self-sampling, which did not correlate to test outcomes.

Increasing duration of the disease and the concomitant presence of anti-SARS-CoV-2-IgG correlated with negative test results in self-collected samples of previously confirmed SARS-CoV-2 positive individuals in line with the clearance of viral load in the course of the disease. Thus, self-sampling should be established at the time of symptom onset.

This study has several limitations. It was conducted at a single center, and the sample size was limited due to the HCP risk exposure. Only 74% of hospitalized participants tested positive in the case of HCP-OPS samples, which might be explained by the fact that initial swabs for routine diagnostics were collected using combined oro- and nasopharyngeal swabs, which are found to be superior to oropharyngeal swabs [16]. Furthermore, the test performed in routine clinical practice was allowed in the previous 48 h and no minimum value of the viral load had been defined for inclusion. Given that anti-SARS-CoV-2-IgG had been detected in 20 participants and symptom onset was reported up to 30 days before presentation, viral clearance may have already occurred in these participants. In addition to oropharyngeal self-sampling, we aimed to investigate the sensitivity and practicability of anorectal self-sampling. SARS-CoV-2 was previously detected in feces and anorectal swabs [17–19]. Anorectal swabs could reduce the exposure of staff and could be more comfortable in participants with a marked gag reflex. However, only 23% of the participants in this study performed SC-ARS. We note that SC-ARS was facultative for participation in our study and concluded that SC-ARS is not well accepted. Although markedly limited by the small sample size, the sensitivity of 6% that was obtained suggests that self-sampling of anorectal swabs is not applicable.

In conclusion, oropharyngeal self-sampling appears to be an applicable alternative testing approach for SARS-CoV-2 diagnosis if certain conditions are met. Based on our findings, we recommend conducting self-sampling immediately after symptom onset, the collection of two distinct samples, and shipping within 48 h. Future studies investigating self-sampling in an outpatient or home-based setting using self-sampling kits are warranted.

## Supplementary Information

Below is the link to the electronic supplementary material.

Supplementary file1 (DOCX 172 kb)

All raw data are depicted in a separate Excel file (XLSX 17 kb)

## Data Availability

All raw data, self-sampling instructions, questionnaires, and answers to half-open question No. 4, as well as comments of the questionnaire are provided in the supplementary information.

## Abbreviations

COVID-19: Coronavirus disease by SARS-CoV-2
HCP: Health Care Professional
HCP-OPS: Health Care Professional-collected oropharyngeal swabs sent for diagnostic without delay
OR: Odds ratio
PCR: Polymerase Chain Reaction
SARS-CoV-2: Severe acute respiratory syndrome coronavirus 2
SC-ARS: Participant self-sampled anorectal swab sent for diagnosis without delay
SC-OPS1: Participant self-collected oropharyngeal swabs sent for diagnosis without delay
SC-OPS2: Participant self-collected oropharyngeal swabs sent for diagnosis after 48 h storage
