# Supplementary material for: Self-sampling versus health care professional-guided swab collection for SARS-CoV-2 testing
Source: Infection. 2021 May 10;49(5):927–34. doi: 10.1007/s15010-021-01614-9 (PMC8107404; doi:10.1007/s15010-021-01614-9)
Supplement: Supplementary file 1 — Supplementary file1 (DOCX 172 kb) [file 15010_2021_1614_MOESM1_ESM.docx]

**Supplementary Information**

**Article title: Self-sampling versus health care professional-guided swab collection for SARS-CoV-2 testing**

**Journal name:**

*Infection*

**Author names:**

Silvia Würstle, Christoph D. Spinner, Florian Voit, Dieter Hoffmann, Svenja Hering, Simon Weidlich, Jochen Schneider, Alexander Zink, Matthias Treiber, Roman Iakoubov, Roland M. Schmid, Ulrike Protzer, Johanna Erber

**Corresponding Author:**

Christoph D. Spinner, PD Dr.

Technical University of Munich, Germany, School of Medicine

University Hospital rechts der Isar

Department of Internal Medicine II

Ismaninger Str. 22, 81675 Munich, Germany

Tel: +49 (89) 4140-4375

Fax: +49 (89) 4140-7555

E-mail: [christoph.spinner@mri.tum.de](mailto:christoph.spinner@mri.tum.de)

**Supplementary Material 1** Self-Sampling instructions in German and English

*Instructions in English are italicized.*


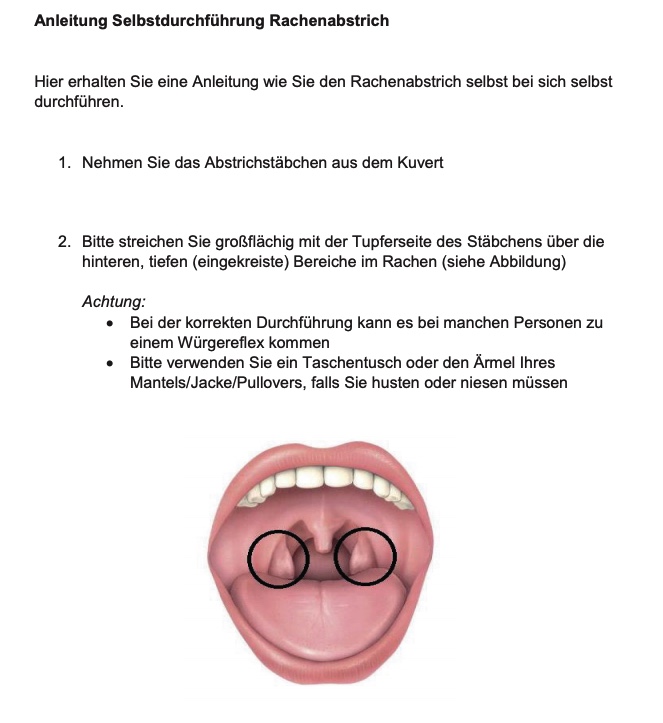


***Instructions for self-collection of an oropharyngeal swab***

*Here, we describe how to collect oropharyngeal swabs.*
*1. Take the swab out of the tube.*

*2. Please use the swab to wipe extensively at the back, deep (encircled) parts of your throat (see picture).*

*Caution:*

- *If performed correctly, the gag reflex is triggered in some people.*
- *Please use a tissue or sleeve to protect your mouth if you need to cough or sneeze.*

**Anleitung Rektalabstrich**

Tipp: Die Abnahme ist leichter, wenn Sie dabei in die Hocke gehen oder einen Fuß auf eine Erhöhung (z.B. Stufe) stellen

(1) Hände waschen

(2) Abstrich-Röhrchen aus dem Set entnehmen und öffnen

(3) Das Stäbchen vorsichtig 2–3 cm rektal einführen, drehen und wieder herausziehen

(4) Das Stäbchen zurück in das Röhrchen Stecken

(5) Hände waschen

***Instructions for the collection of an anorectal swab***

*Hint: It is easier to perform the collection if you squat or put one foot on a step.*

*(1) Wash your hands.*

*(2) Take the sample out of the package and open the tube.*

*(3) Introduce the swab 2–3 cm into the anus, rotate, and drag it out.*

*(4) Put the swab back into the tube.*

*(5) Wash your hands.*

# **Supplementary Material 2** Questionnaire in German and English

# **Welche Probleme traten bei der Selbstdurchführung der Rachenabstriche auf?** Which challenges occure during self-sampling?

- Keine/ *none*
- Schmerzen/ *pain*
- Würgereiz/ *gag reflex*
- Blutung/ *bleeding*
- Ich habe die Gebrauchsanweisung zur selbständigen Durchführung nicht verstanden./ *I did not understand the instructions for self-sampling.*
- Ich war mir unsicher, wo genau im Rachen ich den Abstrich durchführen soll./ *I was not sure where exactly to collect the sample from the throat.*
- Ich konnte das Stäbchen nicht in das Transportröhrchen stecken./ *I failed to insert the swab into the tube.*
- Ich habe Hilfe durch eine andere Person bei der Durchführung gebraucht./ *I needed help from another person to perform self-sampling.*
- Sonstige Probleme (Freitext)/ *other problems (open answer)*

# **Nachdem Sie die Abstriche nun selbstständig durchgeführt haben und Abstriche bei Ihnen durch das medizinische Personal durchgeführt wurden: Welche Art der Durchführung würden Sie grundsätzlich bevorzugen?** *Now that you have self-collected swabs and health care personnel have collected swabs: Which way would you generally prefer?*

- Eine selbstständige Durchführung der Abstriche/ *Self-sampling*
- Eine Durchführung durch medizinisches Fachpersonal/ *Health-care personnel guided sampling*
- Ist mir egal./ *I do not mind.*

# **Können Sie Ihre Wahl begründen (Mehrfachauswahl möglich)** *Can you explain your choice (multiple answers possible)?*

- Die selbstständige Durchführung ist weniger unangenehm./ *Self-sampling is less uncomfortable.*
- Bei der selbständigen Durchführung habe ich weniger Angst./ *Self-sampling is less frightening.*
- Die selbstständige Durchführung ist einfacher./ *Self-sampling is easier.*
- Die selbstständige Durchführung ist zeitsparender./ *Self-sampling is less time consuming.*
- Die Durchführung durch medizinisches Fachpersonal ist weniger unangenehm./ *Health care professional-guided sampling is less uncomfortable*.
- Bei der Durchführung durch medizinisches Fachpersonal fühle ich mich sicherer./ *I feel more secure* with *health care professional-guided testing*
- Bei der Durchführung durch medizinisches Fachpersonal habe ich weniger Angst./ *I am less frightened if health care professionals collect the sample.*
- Die Durchführung durch medizinisches Fachpersonal ist einfacher./ *Health care professional-guided sampling is easier.*
- Die Durchführung durch medizinisches Fachpersonal ist einfacher./ *Health care professional-guided sampling is less time consuming.*
- Andere Gründe (bitte nennen)/ *other reasons (please name)*

# **Können Sie sich vorstellen in der Zukunft Rachenabstriche selbständig, z. Bsp. Zuhause durchzuführen?** *Would self-sampling at home be conceivable for you?*

- Ja, weil: …/ *Yes, because ….*
- Nein, weil …./ *No, because ….*

# **Haben Sie weitere Anmerkungen oder Kommentare?** *Any further notes or comments you would like to make?*

# **Supplementary Material 3** Transcribed answers and comments of patients in German and English translation

# **Welche Probleme traten bei der Selbstdurchführung der Rachenabstriche auf?** *Which problems occur during self-sampling?*

- Sonstige Probleme (Freitext)/ *other problems (open answer)*
- Stäbchen im After verbiegt sich/ *The swab bends in the anus.*
- Das Röhrchen geht schwer auf/ *It is hard to open the tube.*

# **3. Können Sie Ihre Wahl begründen (Mehrfachauswahl möglich)** *Can you explain your choice (multiple answers possible)?*

- Andere Gründe (bitte nennen)/ *other reasons (please name)*
- Unsicherheit von Patienten wie tief/ *patients feel unsure how deep swabs need to be inserted.*
- Egal/ *indifferent*
- Schützt Personal/ *protects health care personnel*

# **4. Können Sie sich vorstellen in der Zukunft Rachenabstriche selbständig, z. Bsp. von Zuhause durchzuführen?** *Would self-sampling at home be conceivable for you?*

- Ja, weil: …/ *Yes, because ….*
- … es unkompliziert und einfach ist./ … *it is straightforward and easy.*
- … es weniger Umstände bereitet/ … *it causes less trouble.*
- … kein Problem/ *… No problems.*
- … unkompliziert und ohne Kontakt/ … *straightforward and contactless*.
- … es ist doch einfach./ *…. it’s easy.*
- Personal entlastet werden kann/ *staff is released.*
- … organisatorisch einfacher./ *simplifies the organization*.
- … erleichtert Arbeit der Ärzte senkt Risiko./ *… simplifies doctors‘ work and decreases the risk.*
- *…* einfach/ … *easy.*
- *…* einfacher/ … *easier.*
- Nein, weil …./ *No, because ….*
- *…* Unsicherheit ob richtig, was man kaputtmachen, immer verkehrtes Ergebnis, weil man‘s nicht richtig macht/ … *uncertainty whether it was done correctly, whether it can be harmful, wrong result if misdone.*
- … ich möglicherweise zu vorsichtig bin und der Rachenabstrich möglicherweise unbrauchbar ist./ … *I might be too cautious, and the swab is thus useless.*
- Zu kompliziert./ *too complicated.*
- … lieber vom Hausarzt./ *… prefer GP.*

# **5. Haben Sie weitere Anmerkungen oder Kommentare?** *Any further notes or comments you would like to make?*

- Nur im Notfall selber machen/ *only in an emergency*
- Guter Sinn/ *useful*

# **Supplementary Table 1** Questionnaire evaluation

| Questionnaire (closed questions) | |
| --- | --- |
| No challenges occurred during self-sampling; n/N, (%) | 28/58 (48%) |
| Gag reflex occurred during self-sampling; n/N, (%) | 28/58 (48%) |
| I was not sure where exactly to collect the sample in the throat; n/N, (%) | 4/58 (7%) |
| I needed help from another person to perform self-sampling; n/N, (%) | 1/58 (2%) |
| I would prefer self-sampling over HCP-guided testing; n/N, (%) | 20/58 (34%) |
| I would prefer HCP-guided testing over self-sampling. | 13/58 (22%) |
| I don’t mind whether the swab is collected by HCP or self-sampling; n/N, (%) | 24/58 (41%) |
| Self-sampling is less uncomfortable than HCP-guided testing; n/N, (%) | 10/58 (17%) |
| Self-sampling is less time consuming than HCP-guided testing; n/N, (%) | 5/58 (9%) |
| Self-sampling is easier than HCP-guided testing; n/N, (%) | 14/58 (24%) |
| HCP-guided testing is less uncomfortable than self-sampling; n/N, (%) | 2/58 (3%) |
| HCP-guided testing is less time consuming than self-sampling; n/N, (%) | 1/58 (2%) |
| HCP-guided testing is easier than self-sampling; n/N, (%) | 6/58 (10%) |
| I feel more secure with HCP-guided testing than with self-sampling; n/N, (%) | 16/58 (28%) |
| Would self-sampling at home be conceivable for you? | |
| Yes; n/N, (%) | 40/58 (69%) |
| No; n/N, (%) | 4/58 (7%) |

HCP: health care professional.

**Supplementary Table 2** Uni- and multivariable analysis of predictive coefficients

Univariable analysis was performed using Fisher’s two-sided exact test on categorial variables and Wilcoxon’s rank-sum test on quantitative parameters. Viral load was measured in copies per milliliter (cps/mL).

CI: confidence interval; HCP-OPS Health Care Professional-collected oropharyngeal swabs sent for diagnostic without delay; SC-OPS1 Participant self-collected oropharyngeal swabs sent for diagnostic without delay; OR: odds ratio.

|  |  |  | **Positive result of SC-OPS1** | **No positive result of SC-OPS1** |  | **Positive result of both HCP-OPS and SC-OPS1** | **No positive result of both HCP-OPS and SC-OPS1** |  | **Concordance between HCP-OPS and SC-OPS1** | **Discordance between HCP-OPS and SC-OPS1** |  | **Difference in viral load of HCP-OPS and SC-OPS1** | |  |
| --- | --- | --- | --- | --- | --- | --- | --- | --- | --- | --- | --- | --- | --- | --- |
|  |  |  |  |  |  |  |  |  |  |  |  |  |  |  |
|  |  |  |  |  |  |  |  |  |  |  |  |  |  |  |
|  |  |  |  |  |  |  |  |  |  |  |  |  |  |  |
|  |  |  |  |  | p-value |  |  | p-value |  |  | p-value | Median | Range | p-value |
| Univariable | **Beginning of symptoms >1 week** | Yes | 10 | 15 | 0.001** | 8 | 17 | 0.001** | 16 | 9 | 0.05223 | 1050 | 0–5070000 | 0.015* |
|  |  | No | 22 | 4 |  | 21 | 5 |  | 23 | 3 |  | 61600 | 0–24400000 |  |
|  |  | OR | OR 0.127 | | | OR 0.118 | | | OR 0.239 | | |  |  |  |
|  |  | [95% CI] | [0.024,0.530] | | | [0.025,0.471] | | | [0.036,1.152] | | |  |  |  |
|  | **SC-OPS1 virus load** | Median |  |  |  |  |  |  | 5050 | 0 | 0.001** |  |  |  |
|  |  | Range |  |  |  |  |  |  | 0-23000000 | 0-9080 |  |  |  |  |
|  | **Gag reflex during self-sampling** | Yes | 17 | 11 | 0.582 | 16 | 12 | 1 | 22 | 6 | 1 | 3300 | 0–2337400 | 0.098 |
|  |  | No | 21 | 9 |  | 18 | 12 |  | 23 | 7 |  | 8940 | 0–24400000 |  |
|  |  | OR | OR 0.667 | | | OR 0.891 | | | OR 1.114 | | |  |  |  |
|  |  | [95% CI] | [0.193, 2.248] | | | [0.275, 2.870] | | | [0.271,4.710] | | |  |  |  |
| Multivariable | **Beginning of symptoms >1 week** |  |  |  | 0.002** |  |  | 0.001** |  |  |  |  |  |  |
|  |  |  |  |  |  |  |  |  |  |  |  |  |  |  |
|  |  | OR | OR 0.111 | | | OR 0.107 | | |  |  |  |  |  |  |
|  |  | [95% CI] | [0.024, 0.403] | | | [0.026, 0.372] | | |  |  |  |  |  |  |
|  | **Gag reflex during self-sampling** |  |  |  | 0.474 |  |  | 0.677 |  |  |  |  |  |  |
|  |  |  |  |  |  |  |  |  |  |  |  |  |  |  |
|  |  | OR | OR 0.616 | | | OR 0.759 | | |  |  |  |  |  |  |
|  |  | [95% CI] | [0.154, 2.259] | | | [0.195, 2.746] | | |  |  |  |  |  |  |

#

a) b)


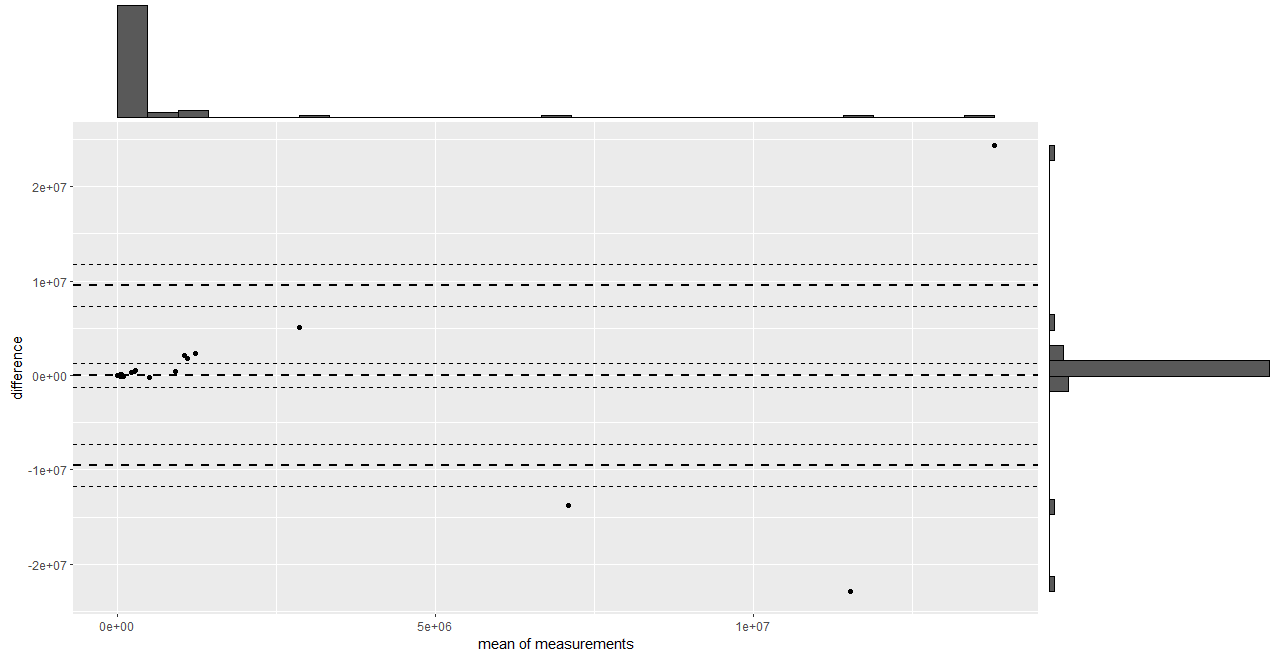

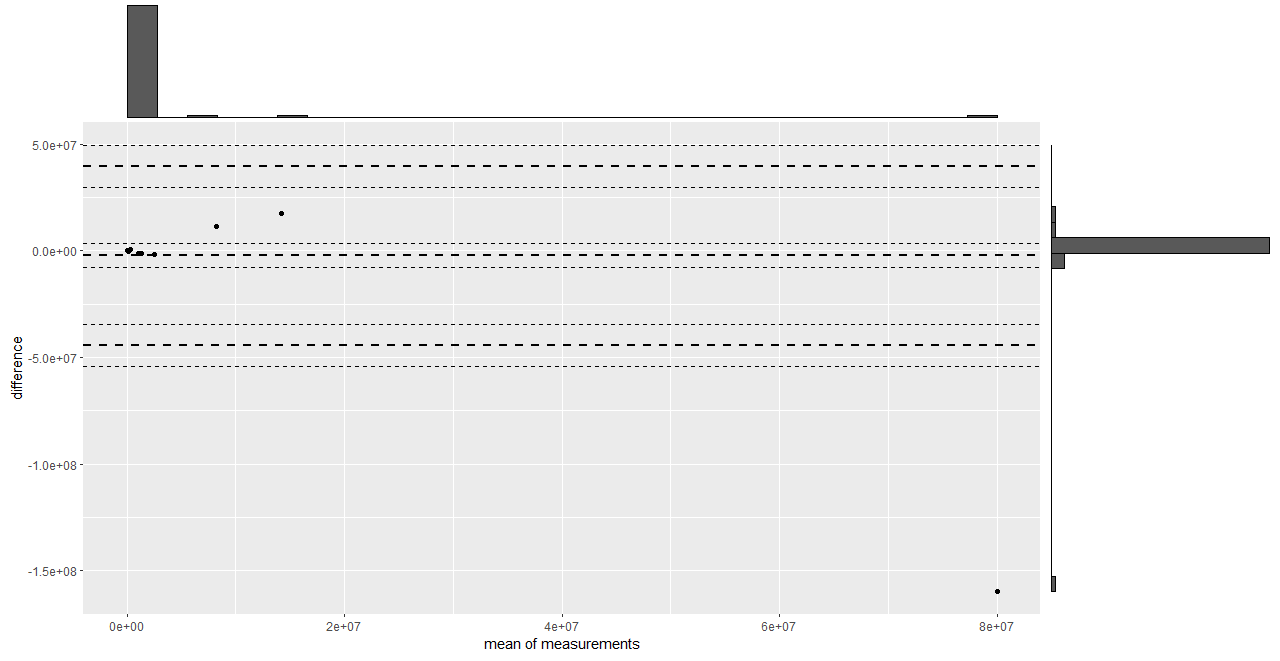


**Supplementary Fig 1** Graphical representation of differences in viral load of different samples

Bland-Altman plots with 95% confidence intervals display differences between viral load in (a) HCP-OPS versus SC-OPS, and (b) SC-OPS1 versus SC-OPS2. HCP-OPS Health Care Professional-collected oropharyngeal swabs sent for diagnostic without delay, SC-OPS1 Participant self-collected oropharyngeal swabs sent for diagnostic without delay, SC-OPS2 Participant self-collected oropharyngeal swabs sent for diagnostic after 48 h storage.

**Supplementary Excel table with raw data**

All “Raw data” are depicted in a separate Excel file.
